# Supplementary material for: From methylation to myelination: epigenomic and transcriptomic profiling of chronic inactive demyelinated multiple sclerosis lesions
Source: Acta Neuropathol. 2023 Jun 7;146(2):283–99. doi: 10.1007/s00401-023-02596-8 (PMC10328906; doi:10.1007/s00401-023-02596-8)
Supplement: Supplementary file 1 — Supplementary file1 (DOCX 100 KB) [file 401_2023_2596_MOESM1_ESM.docx]

**Supplementary Information S1: Guide RNA targeting MBP**


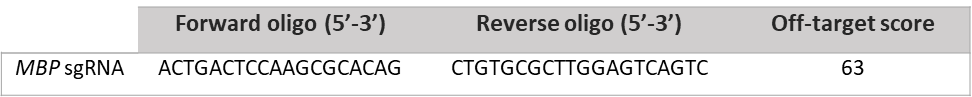


**Supplementary Information S2: Pyrosequencing primer list**


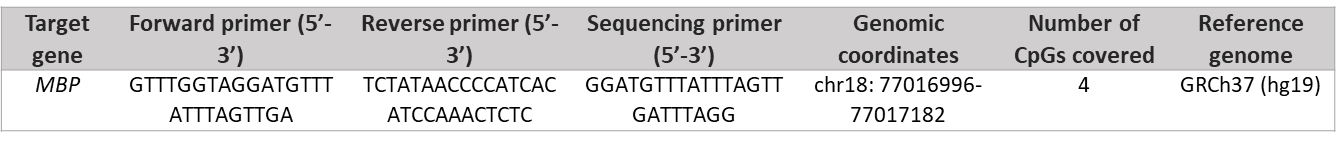


**Supplementary Information S3: qPCR primer list**


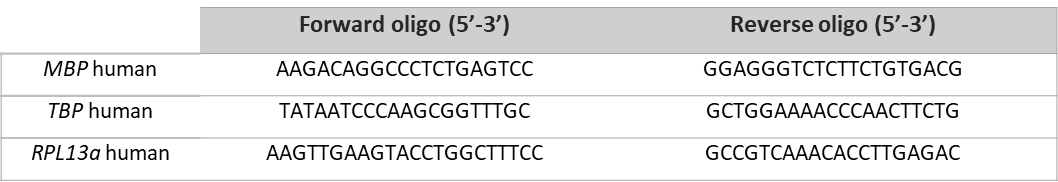


**Supplementary Information S4: Antibody list**


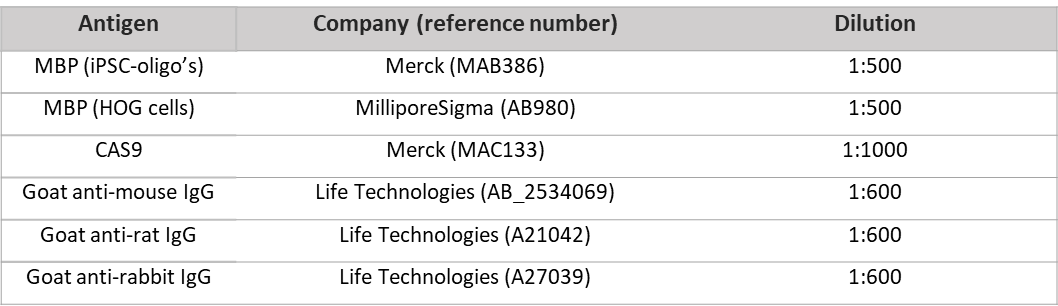


**Supplementary Information S5: Predicted off-target sites of the sgRNA**

**
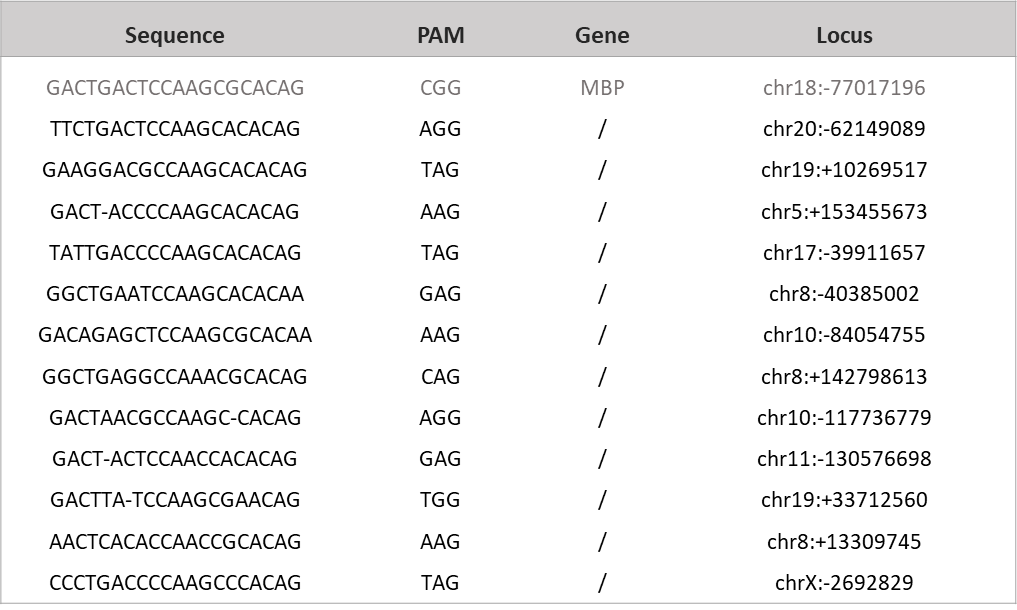
**
